# Improving Light Distribution by Zoom Lens for Electricity Savings in a Plant Factory with Light-Emitting Diodes

**DOI:** 10.3389/fpls.2016.00092

**Published:** 2016-02-09

**Authors:** Kun Li, Zhipeng Li, Qichang Yang

**Affiliations:** ^1^Institute of Environment and Sustainable Development in Agriculture, Chinese Academy of Agricultural ScienceBeijing, China; ^2^Key Lab of Energy Conservation and Waste Management of Agricultural Structures, Ministry of AgricultureBeijing, China

**Keywords:** reserved growing space, principles during transplanting, precise illuminating, zoom, light use efficiency

## Abstract

The high energy consumption of a plant factory is the biggest issue in its rapid expansion, especially for lighting electricity, which has been solved to a large extent by light-emitting diodes (LED). However, the remarkable potential for further energy savings remains to be further investigated. In this study, an optical system applied just below the LED was designed. The effects of the system on the growth and photosynthesis of butterhead lettuce (*Lactuca sativa* var. capitata) were examined, and the performance of the optical improvement in energy savings was evaluated by comparison with the traditional LED illumination mode. The irradiation patterns used were LED with zoom lenses (Z-LED) and conventional non-lenses LED (C-LED). The seedlings in both treatments were exposed to the same light environment over the entire growth period. The improvement saved over half of the light source electricity, while prominently lowering the temperature. Influenced by this, the rate of photosynthesis sharply decreased, causing reductions in plant yield and nitrate content, while having no negative effects on morphological parameters and photosynthetic pigment contents. Nevertheless, the much higher light use efficiency of Z-LEDs makes this system a better approach to illumination in a plant factory with artificial lighting.

## Introduction

A plant factory is regarded as an ideal candidate for precision agriculture (Murase, 2000) due to the observability and controllability of most of its environmental factors, in which artificial lights play an important part. Plant factories with artificial lighting have been applied in many fields (Ting et al., 1993; Watanabe, 2009; Kato et al., 2010, 2011; Shimizu et al., 2011; Christou, 2013; Pamungkas et al., 2014). However, the technology has met difficulties in entering the market due to the large investment in facilities and high operating costs involved (Ohyama et al., 2001; Kwon et al., 2014), precluding large-scale application. With the enormous potential demand, research on the energy consumption of plant factories has become increasingly important and attracted widespread attention.

Several possible solutions for reducing the energy consumption have been studied. Hashimoto (1991) proposed that the computer-integrated plant growth factory for agriculture and horticulture should be expected to be the most effective system in the coming generation. After many years of research, higher levels of environmental management and control techniques were introduced, bringing the plant factory into full play with respect to optimizing the energy efficiency (Seo and Choi, 2011; Ijaz et al., 2012; Wu et al., 2012; Kwon et al., 2014). The power source options of plant factories were also greatly expanded by the development of clean energy technology. Hyun et al. (2014) analyzed various unused energy heat sources such as air source, power plant waste heat, seawater, river, and geothermal heat, and found that the coefficient of performance of the heat pump applied in the study was the highest using power plant waste heat. Waste heat from coal-fired thermal power plants was also introduced to nearby plant factories, reducing fuel oil consumption by 16.05 TJ per year and total CO_2_ emissions by 1204 tons per year (Togawa et al., 2014).

For further energy conservation, electricity consumption should be cut down, especially that used in artificial lighting, which makes up 45% of the total (Ikeda et al., 1992); its use could be reduced by 50% by improving the efficiency of the lighting (Nishimura et al., 2001; Bülow-Hübe, 2008). The application of new types of light source might be the most effective method of energy saving. Previous studies have indicated that among these newly developed artificial lighting technologies, the light-emitting diode (LED) has already been widely used for plant growth (Li and Kubota, 2009; Hogewoning et al., 2010; Li et al., 2010, 2012; Johkan et al., 2012; Samuolienė et al., 2012) for its tailorable spectral composition, wavelength specificity, narrow bandwidth, and foremost, high energy efficiency compared with conventional plant cultivation artificial light sources such as mercury tungsten lamps (Austin, 1965), high-pressure sodium lamps (Sager et al., 1982; Tibbitts et al., 1983), and fluorescent lamps (Andersen, 1986). In recent years, complementary to inorganic LEDs, highly efficient flexible organic light-emitting diodes (OLED) have been characterized by laboratories and found to have an energy efficiency of 30 lm/W at 1000 cd/m^2^, which is two to three times higher than that of common incandescent bulbs (Holst Centre, 2012). Advanced cultivation techniques also play a crucial role in the full exploitation of potential artificial lighting technologies. Yamada et al. (2000) found that stepwise photosynthetic photon flux (PPF) control is a useful method for reducing the electricity consumption of lighting and increasing the electricity utilization efficiency. The lighting consumption has been reported to decrease by 18.4% under a vertical and horizontal movable system, halving the initial light source input while maintaining the yield (Li et al., 2014). Poulet et al. (2014) showed that 50% less energy per unit dry biomass accumulated was used for lettuce crops grown with a targeted LED lighting system.

The elimination of occupied energy on reserved growing space without reducing the photosynthetic yield has drawn our attention as well. Different from the research of Poulet et al. (2014), to find an accurate and effective method to reduce the unnecessary illumination between the gaps between adjacent lettuces in practical application was the focus of our research. On the basis of understanding the transplanting process, two principles were put forward:

(i) Space will always be reserved for plants development until the next transplanting;
(ii) Transplanting will be executed as long as the plant canopies contact with the adjacent leaves.

To explore the potential and effectiveness of this approach, a precise illuminating system involving zoom lenses was built, and its benefits were evaluated by analyzing the electricity consumption of the lighting as well as the plant growth, plant physiology, and phytochemical accumulation of the lettuce grown under different lighting modes. The objective of this research was to develop a lighting system for lettuce production in a plant factory by dynamically focusing the irradiation on the plant canopy, providing a high PPF with low electricity consumption while maintaining the lettuce yield and quality.

## Materials and Methods

### Plant Materials and Growth Conditions

Butterhead lettuce seeds (Flandria RZ, Rijk Zwaan, De Lier, The Netherlands) were sown in a plastic seedling tray (57 cm × 23.5 cm × 4 cm) and germinated in a 50 m^-2^ computer-controlled fully closed plant factory at the Chinese Academy of Agricultural Science (CAAS), Beijing, China (latitude 39°57′40.2″N, longitude 116°19′34.6″E) under dim LED light (50 μmol⋅m^-2^⋅s^-1^) and irrigated with tap water once per day. Approximately 15 day after sowing, 16 uniform seedlings were transplanted onto cultivation boards (polyethylene, 68 cm × 72 cm × 1.4 cm, 32 plants/m^2^) and were cultivated with the deep flow technique (Hu et al., 2008) for 25 day under a 16-h photoperiod on a 24-h light/dark cycle. The air temperature measured at the top of the canopy was maintained at 23 ± 0.5°C during the daytime and 20 ± 0.5°C at night. The relative humidity was 60 ± 5%, and the concentration of carbon dioxide was kept at 400 ± 10 ppm. Modified Hoagland nutrient solution [(±SE) pH6.3 ± 0.1, EC 1.6 ± 0.2 mS⋅cm^-1^] was used, and half dose nutrient solution (EC 1.0 ± 0.2 mS⋅cm^-1^) was employed during the seeding stage. The air temperature and humidity were measured twice per day, and the parameters of the nutrient solution were monitored daily.

### Light Treatments

**FIGURE 1:**
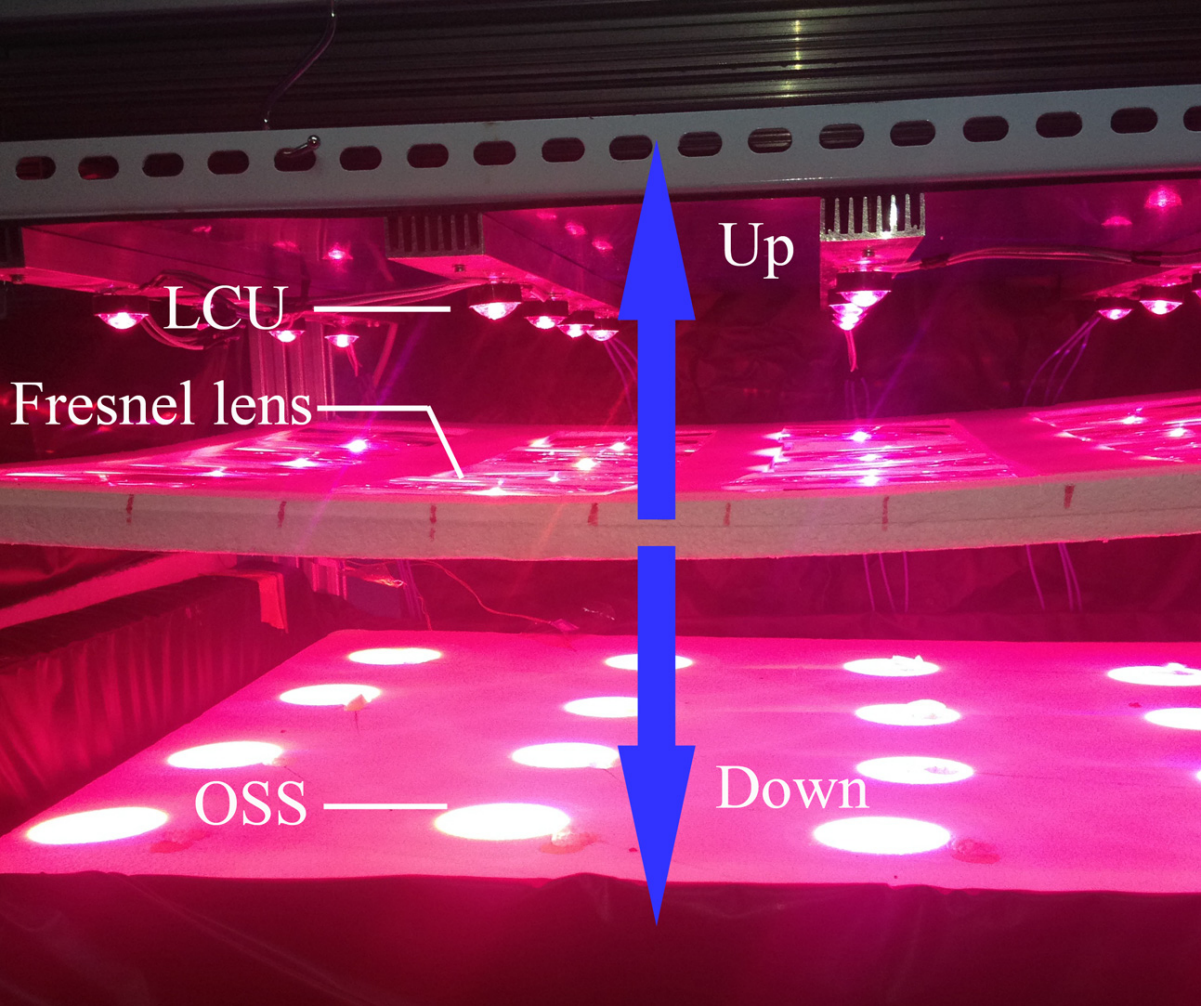
**The precise illuminating system used in this study.** The multi-chip light- emitting diode – convex lens units (LCUs) were located 30 cm away from the seedlings as the light sources. The Fresnel lens array was located between the LCUs and the seedlings, while able to be moved in the directions of the blue arrows to manually adjust the diameters of the orbicular spectrum spots (OSSs).

**FIGURE 2:**
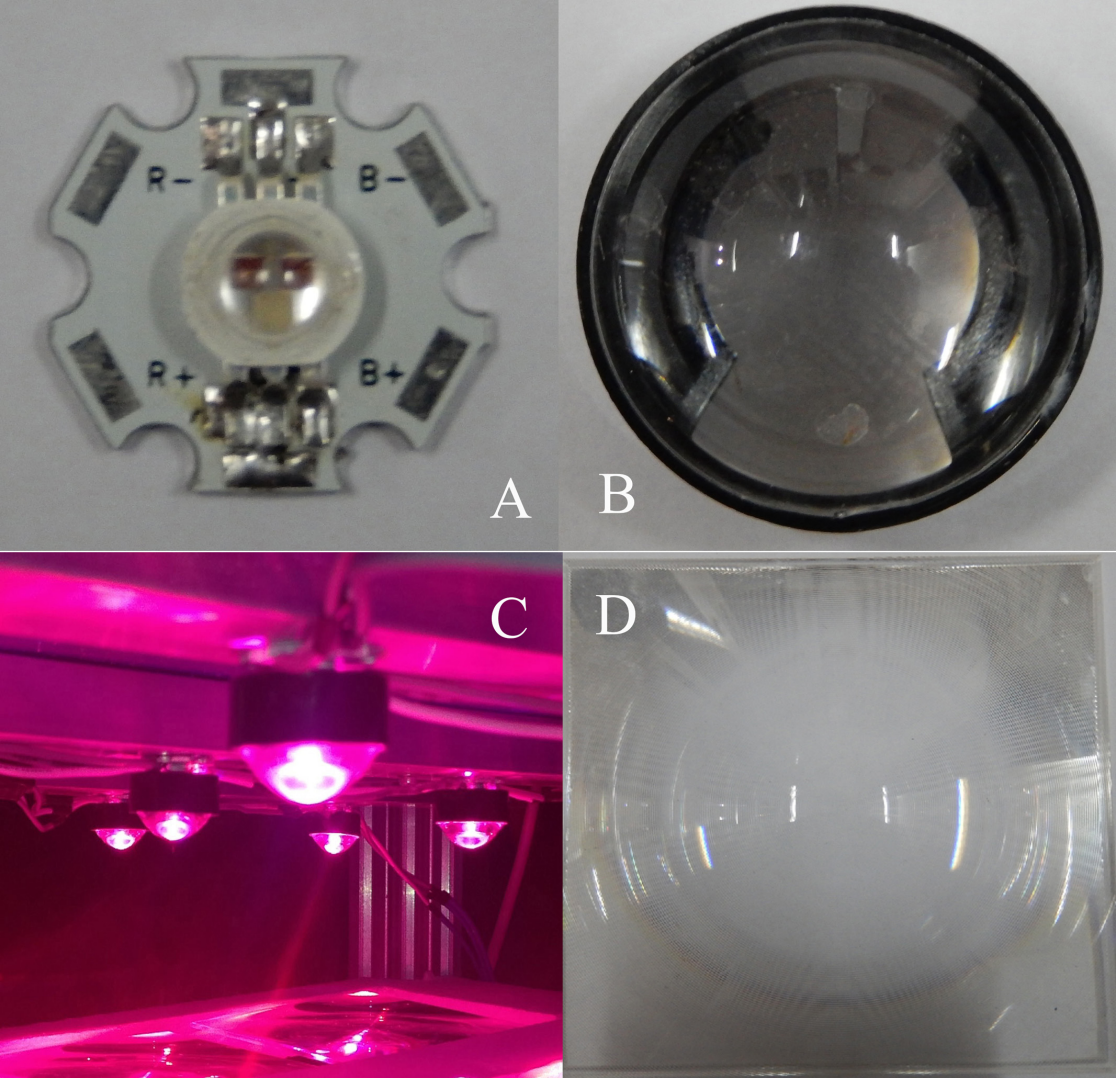
**Multi-chip light-emitting diodes (LEDs) **(A)** have three chips (two red chips and one blue chip) to satisfy the spectrum demand of the lettuce; each chip’s rated power is 3 W.** Combined with the convex lens **(B)**, they form the multi–chip LED – convex lens unit (LCU, **C**), hanging 30 cm over the top of the cultivation boards to accumulate the photosynthetic quantum as much as possible to irradiate the Fresnel lens **(D)**, which focuses the photosynthetic quantum again to illuminate the plants.

**FIGURE 3:**
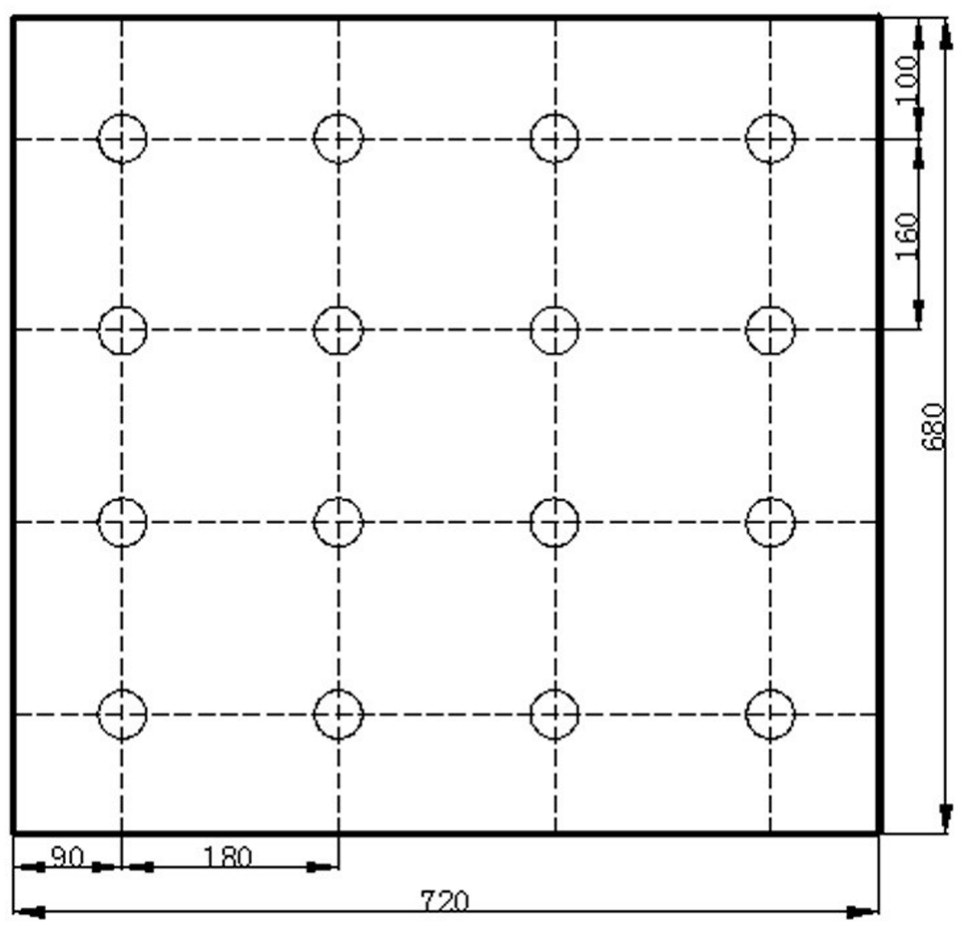
**The position arrangement of the multi–chip LED–LCUs over the cultivation board, indicating the allocation of positions for lettuce seedlings.** All dimensions were in millimeters.

A precise illumination system was built (**Figure 1**) that was composed of 16 multi-chip LEDs (**Figure 2A**, WenLiang Electronics CO., Shenzhen, China). Two red (peak at 630 nm) chips and a blue (peak at 460 nm) chip were fixed on strip form aluminum heat sinks and located right above each plant to form a lighting array (**Figure 3**, 72 cm × 68 cm) as the light source of the zoom LED treatment (Z-LED). The lighting array was placed horizontally 30 cm above the seedlings inside the plant factory to achieve the illumination schedule described below. The rated power for each chip is 3 W, while the output power can be regulated individually by adjusting the current and voltage output of the alternating current to direct current power source (DPS-3005D; ZhaoXin Electronic Instrument Equipment Co., Shenzhen, China) connected to each LED chip. As the optical accessories, 16 convex lenses (**Figure 2B**, 24.50 mm × 14.00 mm, transmittance 93%, HengZheng Optics Technology Co., Ltd., Dongguan, China) were tightly attached to their corresponding LEDs. Directly below the multi-chip LED – convex lens unit (LCU, **Figure 2C**), 16 Fresnel lenses (**Figure 2D**, 120 mm × 120 mm, transmittance 95%, YuYing Optical Instrument CO., LTD, ShanDong, China) were fixed in a horizontal plane frame structure (high-density foam polystyrene), which could be manually moved vertically along the axial direction of the LCU (**Figure 1**).

The photosynthetic quantum in the Z-LED was accumulated by the LCUs as much as possible before irradiation onto the plant canopy, while the diameters of the orbicular spectrum spots (OSS) were adjusted by manually regulating the distance between the Fresnel lens frame and the LCU to adapt the dimensions of the plant canopy (**Figure 1**).

Different from the Z-LED treatment, four custom-manufactured LED panels (60 cm × 25 cm × 1.2 cm; FHT Co., Shenzhen, China) with red (peak at 630 nm) and blue (peak at 460 nm) LEDs were used as the main light source in the conventional non-lens LED (C-LED) treatment and were placed horizontally 30 cm above the seedlings inside the plant factory to achieve the illumination.

**FIGURE 4:**
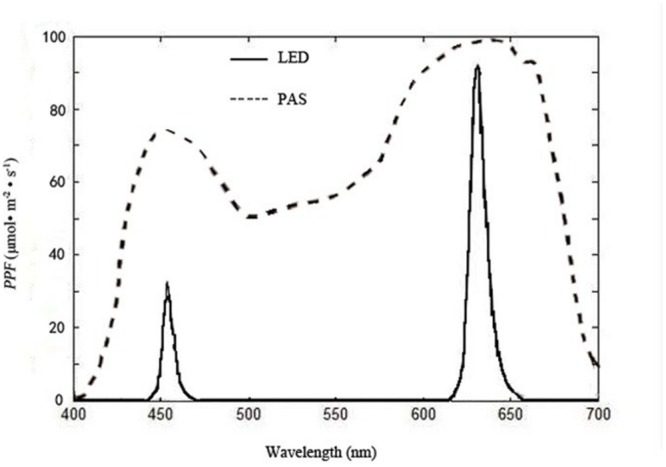
**Spectral distributions of light from multi-chip LEDs, measured with a fiber optic spectrometer (AvaSpec-2048; Avantes, Apeldoorn, The Netherlands), and plant absorption spectrum (PAS) reported by Sager et al. (1982); PPF = photosynthetic photon flux**.

The red–blue ratio (R/B) for both treatments was kept at 8:1 (Wen, 2009). Each treatment covered 0.5 m^2^ (completely covering the growing area of the plants below) was carefully isolated by black films to prevent light contamination from each other during the experiment. The PPF on the plant canopies of the treatments were kept at 70 μmol⋅m^-2^⋅s^-1^ by adjusting the luminous intensity of the individual LEDs for the first 10 days, allowing it to rise to 120 μmol⋅m^-2^⋅s^-1^ for the following 15 days. The illumination time was 16 h per day (0800 to 2400 HR). The spectral energy distribution scans were recorded at 400 to 800 nm with 2-nm steps of the LEDs (**Figure 4**) with a calibrated fiber optic spectrometer (AvaSpec-2048; Avantes, Apeldoorn, The Netherlands) placed horizontally under the light sources used for the experiments.

### Measurements

Once the seedlings were transplanted onto the cultivation board, the diameters of the plant canopies were measured daily. A vernier caliper was employed to gage the length between the central leaf and the edge of the projection of the longest leaf, representing the maximum radius of the OSS of the plant.

The ambient temperatures of the growth were recorded using a data acquisition system (Model CR1000, Campbell Scientific, Inc., North Logan, UT, USA) at 10 min intervals. The temperature was measured using Type T thermocouples (accuracy was ±0.2°C). All thermocouples were measured for 25 days at each point and kept immobile. The leaf temperature was measured by thermocouple, following (Luján et al., 2009).

The plant photosynthetic data were measured on the last day of the growth. The leaf photosynthesis was measured with a portable gas exchange device equipped with a leaf chamber fluorometer (LI-6400; LI-COR). Measurements were carried out between 0900 and 1200 HR, and the samples of two treatments were conducted alternatingly. For the measurements, six mature leaves from different plants were selected. The PPF was 120 μmol⋅m^-2^⋅s^-1^; the measurements were taken when the photosynthesis rate reached steady state (after approximately 10 min). The vapor pressure deficit in the leaf chamber was maintained below 1 kPa; the leaf temperature and CO_2_ concentration in the measurement chamber were maintained at 20°C and 400 ppm, respectively.

Twenty-five days after transplanting, the plants were harvested to measure their growth and to analyze their phytochemical concentrations. The fresh weight (FW), dry weight (DW), plant height, number of leaves, leaf length, leaf area, and leaf chlorophyll (Chl) concentrations were measured after harvest. The leaf areas were measured by the cut-paper weighing method, following Minaxi and Saxena (2010). The plant height, leaf length and leaf width were measured using a vernier caliper (Wu et al., 2007). The leaves were packaged in plastic bags and preserved in an ultralow temperature refrigerator, prepared to be homogenized and used in the determination of ascorbic acid, protein, nitrate and soluble sugar concentrations.

An electricity meter (LCDG-ZJ120-01; LiChuang Science and Technology Co., Laiwu, China) was employed to measure the electricity consumption of the illumination (the energy costs for cooling, ventilation and the recirculation of the nutrient solution were not accounted for in our experiments). The light utilization efficiency [LUE (grams per kilowatt hour)] of each treatment was determined [LUE = leaf FW (grams per plant) ⋅ 32 plants/m^2^/electric energy consumption of lighting (kilowatts per hour)].

The weighed fresh leaf tissue (2 g) was extracted in 96% alcohol/water (v/v) (50 ml for each gram). The extract was centrifuged (3K15; Sigma Laborzentrifugen, Osterode am Harz, Germany) at 10,000 *g* for 10 min. The supernatant was separated, and the absorbance was read at 400–700 nm on a spectrophotometer (UV-1800; SHIMADZU Co., Kyoto Japan) at wavelengths of 663 nm (A663) and 646 nm (A646), respectively. The chlorophyll a (Chl a) and chlorophyll b (Chl b) concentrations were measured by spectrophotometry and calculated according to the following equations of Lichtentaler and Wellburn (1983): Chl *a* = (12.21 × A663–2.81 × A646) × 20/1000/2 and Chl b = (20.13 × A646 –5.03 × A663) × 20/1000/2.

The nitrate content was determined as described by Cataldo et al. (1975). Freeze-dried samples (2 g) were homogenized in 15 ml water and buffer solution to 25 ml volume. The homogenates were filtered, and the filtrates were centrifuged at 10,000 *g* for 15 min. The supernatants were decanted and saved for analysis. A quadruple volume of salicylic acid [5% (w/v)] was mixed, and the pH value was controlled to above 12 by the addition of 9.5 ml 8% sodium hydroxide. The liquid supernatant was measured by spectrophotometry at a 410 nm wavelength to determine the nitrate content.

Protein solutions were prepared in 0.15 M sodium chloride. Freeze-dried samples (0.3 g) were extracted in 5 ml solvent and then centrifuged at 3,000 gn for 10 min. Coomassie brilliant blue G-250 [(5 mL, 0.01%(w/v)] was added into 1 ml of the supernatant and was shaken frequently for 2 min before the spectrophotometric detection at 595 nm wavelength. The protein content was measured following Bradford (1976).

Soluble sucrose content was determined as described by Beck and Bibby (1961). Freeze sample (0.3 g) were extracted in 10 mL of water, 0.5 ml anthrone ethyl acetate, and 0.5 ml concentrated sulfuric acid before incubated in a boiling water bath for 10 min. After constant volume to 25 ml, samples were read in the spectrophotometric at 630 nm wavelength. All the phytochemical concentration experiments were repeated 5 times for error reduction.

### Experimental Design and Statistical Analysis

The experiment was performed twice. Statistical analysis was performed using SAS software (version 9.2; SAS Institute, Cary, NC, USA). Variance analyses within treatments were used, and different letters within the column indicate significant differences at *P* ≤ 0.05 according to the least significant difference test.

### Experiment 1: Establishing Lettuce Growth Curve at Experimental Irradiance

**FIGURE 5:**
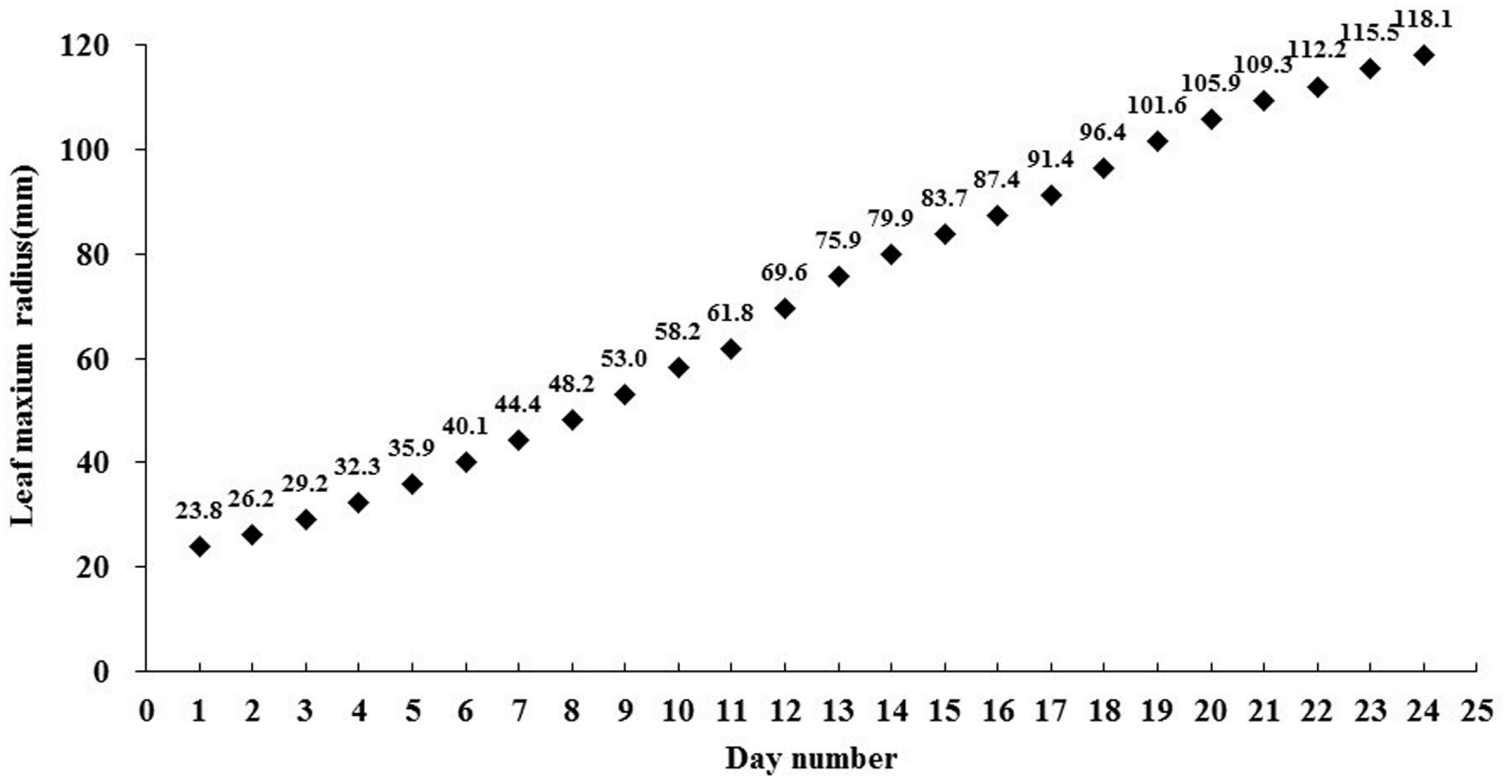
**The curve of the average leaf maximum radius from 36 lettuce plants during 25 days of growth under the conventional non-zoom LED case**.

For the growth curve experiment, 36 plants were cultured under such circumstances as described before in Plant materials and growth conditions while exposed under the light environment described above in Light treatments by C-LED. To maintain the designed light quality and PPF under the constantly changing canopies, the luminous intensity of the individual LEDs were set up every 5 days by adjusting the output of the power supply. The diameters of the lettuces were examined every day for 25 days from seedling to final harvest. We can obtain the detailed diameter of the lettuce in **Figure 5** to find the reasonable size of the OSS. We also calculate the theoretical energy savings.

### Experiment 2: Growth and Energy Consumption Comparison Between Z-LED and C-LED

According to the preliminary experiment’s result, an appropriate Fresnel lens array was employed in Z-LED, combined with suitable LCUs (**Figure 2C**), so that the OSS that fell on the plant canopy could be adjusted to match the diameter of the growing plants by manually adjusting the distance between the Fresnel lens array and the multi-chip LEDs every day. The diameter of the OSS decreased (zoom out) with the increasing of the distance, while the diameter increased (zoom in) with the decreasing of the distance. The physiological and biochemical parameters were determined by using the methodology described above in Measurements. To avoid the unwanted variation of the light environment parameters with the growth of the diameter and height of the plants in both treatments, the light sources were located at an identical height (30 cm) above the cultivation boards, while the luminous intensity of the individual LEDs and the diameter of OSS were adjusted by regulating the output of the power supply and the distance between the Fresnel lens array and LCUs, respectively.

### Experiment 3: Effects of Z-LED on the Ambient Temperature of Plant Growth

Considering the tremendous diversities between Z-LED and C-LED, the environmental parameters, more specifically, a temperature of three sampling point (plant leaf, nutrient solution, and gap on cultivation board between OSS) arrangement was designed for both treatments. The temperatures from all six sampling points were monitored through the entire experiment. The data acquired at 1200 HR everyday were used for analysis. This was chosen because the sampling points had been warmed up by the light source irradiation for 4 h, gradually reaching the equilibrium state and were also not affected by the nutrient solution circulation.

## Results

### Performance Characteristics and Electric-Energy Consumption of the Lighting Systems

Based on the growth surveillance of the lettuce under the scheduled light environment, the radius curve of projection area of plant on the cultivation board was established (**Figure 5**), which indicated the expanded tendency that the OSS should follow. During 25 days’ growth, the leaf radius increased at a varying velocity from 23.8 to 118.1 mm. With the closed observation during the experiment, 14 days after transplanting, when the radius reached 79.9 mm, the contiguous OSSs were tangent to each other during expansion. The optical component parameters of the precise illumination system were determined. High-power (9 W) multi-chip LEDs were employed, and the heat sinking was fully considered. The convex lenses were confirmed to possess an emission angle of 60°. The optical characteristics of the Fresnel lenses used were a focal length of 100 mm, a focus diameter of 4 mm, and dimensions of 120 mm × 120 mm.

As a result, when used as an alternative to conventional LED lights, the Z-LED consumed much less (52.06%) electricity (**Table 1**), but, the plant yield decreased significantly (**Table 2**). Despite this, by benefitting from the excellent energy saving effect, the Z-LED achieved a 55.64% increase in LUE relative to the C-LED (**Table 1**), allowing the plants to obtain the desired illumination with minimal energy consumption.

**Table 1:** Electricity consumption of lighting, plant yields and light utilization efficiencies (LUEs) of lettuce under zoom lens lighting-emitting diode (Z-LED), and conventional non-lens LED (C-LED).

Energy parameters	Z-LED	C-LED
Electricity consumption (kWh/m^2^)	23.73	49.51
Plant yields (g/m^2^)	1130.72 ± 12.19b	1496.93 ± 25.04a
LUEs (g/kWh)	94.38 ± 1.02a	60.64 ± 1.03b

*The electricity consumptions were recorded with an electricity meter. Light utilization efficiencies were estimated by dividing the yield (g/m^-2^) by the electricity consumption of lighting per area. Data were analyzed by analysis of variance, and different letters within the column indicate significant differences at *P* ≤ 0.05 according to the least significant difference test.*

**Table 2:** Lettuce fresh weight (FW), dry weight (DW), leaf area, plant height, true leaf number, leaf length and leaf width for lettuce under Z-LED and C-LED.

Growth parameter	Z-LED	C-LED
Leaf FW (g)	37.68 2.04b	45.23 5.69a
Leaf DW (g)	1.31 0.21b	1.89 0.45a
Leaf area (cm^2^)	1.22 10ˆ3 185.52a	1.22 10ˆ3 150.73a
Plant high (mm)	68.56 11.62a	75.01 9.06a
True leaf number	29.10 1.52a	28 2.45a
Leaf length (mm)	142.87 4.35a	141.57 4.12a
Leaf width (mm)	83.85 6.72a	84.65 7.72a

*Data were analyzed by analysis of variance, and different letters within the column indicate significant differences at *P* ≤ 0.05 according to the least significant difference test.*

### Influence on Temperature

**FIGURE 6:**
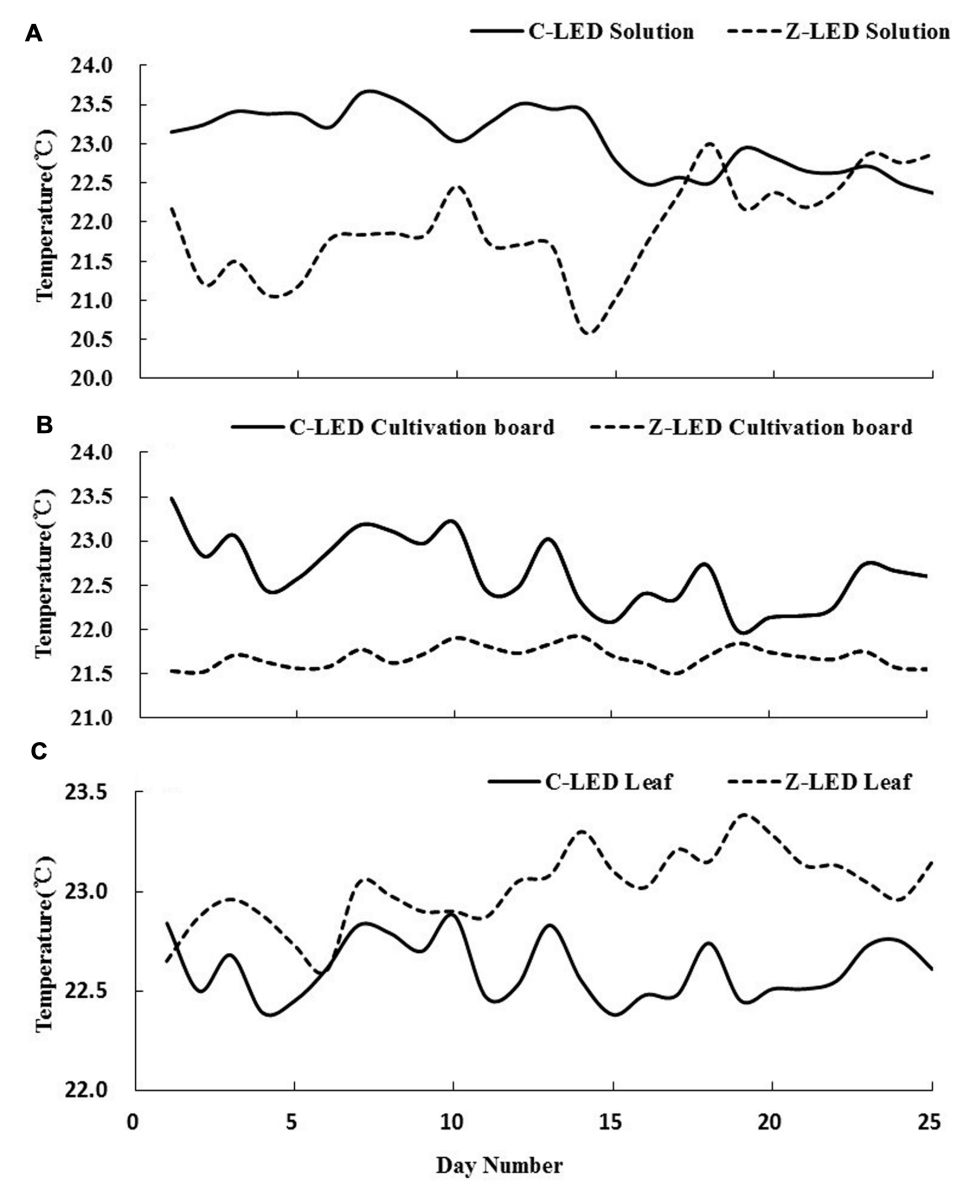
**Temperature of nutrient solutions **(A)**, cultivation boards **(B)**, and leaves **(C)** from zoom lens lighting-emitting diode (Z-LED) and conventional non-lens LED (C-LED), measured from transplanting to harvest (25 days).** All data were acquired from 1150 to 1210 HR every day.

There were significant differences in temperature on the plant leaf, cultivation board, and nutrient solution between two treatments. As shown in **Figure 6A**, there was a large gap in the temperature of the nutrient solution before day 10. The temperatures were approximately 23 and 21.5°C in the C-LED and Z-LED, respectively. Subsequently, the curve rapidly coincided at 22.5°C just after day 10 and maintained this level until the final day. The cultivation board temperature of the Z-LED was 21.5°C throughout the entire experiment, while a descent could be observed from approximately 23 to 22.5°C in the C-LED treatment after the tenth day (**Figure 6B**). Unlike the tendency of the data in the cultivation board and nutrient solution, the canopy temperature of Z-LED (**Figure 6C**) notably increased compared with the C-LED, despite the massive amount of heat energy that had been removed from its system. In addition, the Z-LED canopy temperature manifested a further elevation to approximately 23.5°C on the 10th day, while its counterpart was maintained at 22.5°C throughout the whole experiment period.

### Plant Growth

The lettuce growth was significantly affected by the different light modes in the treatments (**Table 2**). However, unexpectedly, notable negative effects were observed in the Z-LED treatment, leading to 23.07 and 30.68% decreases in the leaf FW and DW, respectively. The difference is not evident in physiological parameters such as plant height, true leaf number, leaf area, leaf length, and leaf width. The C-LED treatment exhibits an apparent improvement in all photosynthetic parameters compared with that of the Z-LED (**Table 3**), with increases of 68.01, 75, 60, and 12.16% for net photosynthesis, stomatal conductance, intercellular CO_2_ concentration, and transpiration rate, respectively.

**Table 3:** Net photosynthesis (Pn), stomatal conductance (Cond), transpiration rate (Tr), and intercellular CO_2_ concentration (Ci) data subjected to the zoom lens lighting-emitting diode (Z-LED) and conventional non-lens LED (C-LED).

Photosynthesis parameter	Z-LED	C-LED
Pn (μmol CO_2_m^-2^s^-1^)	2.72 0.46b	4.57 0.43a
Cond (mol H_2_Om^-2^s^-1^)	0.04 0.01b	0.07 0.02a
Tr (mmol H_2_Om^-2^s^-1^)	0.30 0.08b	0.48 0.09a
Ci (μmol CO_2_mol^-1^)	265.43 11.96b	297.70 24.54a

*Data were analyzed by analysis of variance, and different letters within the column indicate significant differences at *P* ≤ 0.05 according to the least significant difference test.*

### Phytochemical Accumulation

The phytochemical concentrations in the lettuce leaves were significantly affected by the different light treatments (**Table 4**). The focused lighting in the Z-LED revealed a tremendous enhancement effect on the accumulation of soluble sucrose, leading to a remarkable increase of 171.55% in its concentration compared with that of the C-LED, while the nitrate content in the Z-LED treatment was decreased by 23.92%. The different illumination modes seemed to have little influence on the production capacity of the photochemical products, as similar contents of protein, Chl a, and Chl b were observed in both treatments.

**Table 4:** Concentrations of sucrose, nitrate, protein, chlorophyll a (Chl a), and chlorophyll b (Chl b) of lettuce under Z-LED and C-LED.

Physiological parameter	Z-LED	C-LED
Sucrose (mg/g)	34.08 10.48a	12.55 8.59b
Nitrate (μg/g)	177.81 68.06b	233.73 53.38a
Protein (mg/g)	0.26 0.01a	0.26 0.01a
Chl a (mg/g)	0.40 0.01a	0.43 0.05a
Chl b (mg/g)	0.10 0.01a	0.10 0.02a

*Data were analyzed by analysis of variance, and different letters within the column indicate significant differences at *P* ≤ 0.05 according to the least significant difference test.*

## Discussion

### Performance Characteristics and Electric Energy Consumption of the Lighting Systems

The tangency of the contiguous OSSs suggests that leaf overlapping was about to be observed, thereby reducing the energy efficiency of the system. Before that, a large number of reserved growing spaces were energy occupied without photosynthetic yield, representing considerable potential in improving energy efficiency. To provide the plant canopy with the designed light intensity (120 μmol⋅m^-2^⋅s^-1^) when the energy of single LED was dispersed with the OSS expansion, a higher single LED power was required to achieve an identical PPF to the C-LED. In this study, 9 W seemed to be the minimum power supply to meet the needs of the experiments, especially for the fully grown lettuce. Serving as one of the LCU components, convex lenses were used to gather light by narrowing the emission angle of the LEDs. The selected luminous angle (60°) ensured that all focused light fell on the Fresnel lenses, even though the distance between them was maximized to create a small-size OSS on the cultivation board for the seedlings. For the Fresnel lenses, the optical parameters described above were essential characteristics, with which the Fresnel lens array is capable of providing a continuously variable OSS size by adjusting the distance away from the LCUs.

For the electricity saving property, the photons dissipated due to the much wider luminous angle of the C-LEDs, especially of the LEDs that were located on the edge of the cultivation system, being focused onto the plant canopy in the Z-LED treatment. Furthermore, the improved light distribution technology eliminated the extraneous irradiation used to illuminate the non-canopy area. However, this technology, which had the best energy savings effect before the flourishing stage of the lettuce, subsequently declined along with the growth (**Table 5**).

**Table 5:** Piecewise energy consumption under Z-LED and C-LED, recorded by an electricity meter every 5 days.

Piecewise energy consumption (kWh)	Z-LED	C-LED
1–5 days	0.66	3.84
5–10 days	0.78	3.84
10–15 days	2.21	5.52
15–25 days	8.32	11.04
Total	11.98	24.24

The seedlings were transplanted at the density for fully grown lettuce. The application of this technology in practical production, in which the transplanting process would be done approximately three times, could achieve identical performance for conservation as described in this study as long as the two principles of transplanting described earlier are followed.

### Influence of Temperature

The Z-LED significantly decreased the amount of energy entering the plant during growth by 52.06%, thereby having an obvious effect on the temperature of the growing environment. The 10th day was the key inflection point for both treatments at all inspection sites because of the elevation of the PPF from 70 to 120 μmol⋅m^-2^⋅s^-1^ on the plant canopy according to the experiment design, which introduced more heat into both treatments. Due to the use of this technology, much less thermal energy was generated to achieve the PPF. Thus the temperatures of the C-LED were always higher than those of the Z-LED on the cultivation board and nutrient solution for the entire growth phase (**Figure 6**).

**Figure 6B** indicates that the cultivation boards of the Z-LED treatment were not completely exposed under the light sources, and accordingly, the temperature was stable at a low level for the whole period. The irradiation that was supposed to be distributed on the cultivation boards below the C-LED was blocked by the rapidly growing leaves, resulting in a decline of the temperature even though the PPF was higher than before.

As shown in **Figure 6A**, the solution of the C-LED treatment was warmed up by the significant excess energy from the cultivation board through the thermal transition effect. Along with the lowering of temperature on the cultivation board as described before, the solution temperature also showed a tendency to decrease. With the rising of the PPF after day 10, the temperature of the Z-LED went up under the heating effect.

An interesting phenomenon occurred during the temperature monitoring of the plant leaves (**Figure 6C**). The Z-LED treatment presented a higher temperature with much less energy intake. The reason might be the improvement of photosynthesis in the C-LED treatment due to the relatively higher temperature of the growth environment (Berry and Bjorkman, 1980). As described before, all photosynthetic parameters of the C-LED treatment were significantly higher than those of the Z-LED treatment (**Table 3**). More specifically, the transpiration rate was 60% higher in the C-LED treatment than in the Z-LED treatment, which led to a conspicuous reduction of the leaf epidermis temperature (Gates, 1964; Pallas et al., 1967).

### Plant Growth

The lettuce growth was significantly affected by the photosynthesis (Nelson, 1988). Our study showed that the much higher photosynthesis in C-LED, benefitting from the excess irradiation, would result in a large increase in plant yield. The reason might be the light brought in by reflection from the cultivation board where the lettuce was grown (Poulet et al., 2014). However, the morphological parameters of the lettuce were more likely to be affected by the light quality and intensity (Tei et al., 1996; Kitaya et al., 1998; Dougher and Bugbee, 2001; Folta, 2004; Kim et al., 2004; Folta and Childers, 2008), which were set to be consistent within treatments. Contributions to the much higher yields in C-LED might be from the relatively lower root zone temperature (Challa et al., 1995), combined with the thicker leaves when grown at a cooler leaf temperature (Wolfe, 1991).

As one of the most important environmental factors, temperature plays a very important role in plant growth. Berry and Bjorkman (1980) have shown that raising the temperature would contribute to the improvement of photosynthesis in an appropriate range of 20–30°C. He et al. (2001) indicated that a higher temperature in the root zone could improve the photosynthesis and stomatal conductance, consistent with Gosselin and Trudel (1986). Agreeing with them, in our study, the relatively high temperature of the C-LED on the cultivation board and in the nutrient solution results in outstanding improvements in both photosynthesis and plant yield compared to Z-LED, even though the dissolved oxygen value would be lowered (Goto et al., 1996; Yoshida et al., 1997). Furthermore, a high transpiration rate had been reported to be helpful in the cooling of the leaf epidermis (Gates, 1964; Pallas et al., 1967). The higher transpiration rate in the C-LED treatment would decrease the leaf ambient temperature, which would be of great help to improve the yield in the C-LED treatment.

To provide a more complete system for applying this technology to practical applications, the photosynthetic and plant growth performance must be improved to overcome the described drawbacks while still maintaining the energy-saving and high-LUE features of Z-LED. The temperature of the growth environment, firstly, could be slightly increased through the reduced use of air conditioning in the facility, while achieving a further energy saving effect because most of the air conditioning was employed to lower the temperature. Moreover, a growth promotion spectrum such as far-red could be applied in the light sources to enhance the nutritional value and growth of the plants (Li and Kubota, 2009) in appropriate ways. In addition, a significantly higher PPF could be achieved to improve photosynthesis and plant growth, not only by shortening the distance between the light sources and plant canopies (Li et al., 2014) but also by consuming more energy at the expense of the reduction in LUE.

### Phytochemical Accumulation

The phytochemical concentrations in the leaves were also affected by photosynthesis. On account of the increased leaf temperature in the Z-LED, the respiration rate increased considerably (Bolstad et al., 2003), while accelerating the transformation of carbohydrates into sucrose (Williams and Farrar, 1990). To compensate for the shortage of carbohydrates, as was reported by Blom-Zandstra and Lampe (1985), nitrate may serve as the osmoticum instead of sugars in suboptimal photosynthesis, which is the probable cause of the decreased concentration of nitrate in Z-LED. The higher intercellular CO_2_ concentration of the C-LED also played a positive role in the accumulation of nitrate in the C-LED (Kaiser and Förster, 1989; Kaiser and Brendle-Behnisch, 1991). Photosynthetic pigments such as Chl a and Chl b might be much more sensitive to light parameters (Senger, 1982; Kurilčik et al., 2008; Poudel et al., 2008; Li and Kubota, 2009; Li et al., 2012). In this research, we demonstrated that when the parameters are at the same level, Chl a and Chl b have no significant difference from each other, although the photosynthesis has much difference.

## Conclusion

The irradiation on a reserved growing space occupied a large amount of energy, which could be cut off by employing precise illumination before and after transplanting. Precise illumination could reduce electricity use by 52.06% and achieve a 55.64% increase in LUE, while bringing considerable economic benefits. Interestingly, the lower temperature in the growth environment induced by the energy reduction imposed adverse effects on the photosynthesis, reducing the yield significantly. Several countermeasures have been presented and remain to be tested. During the practical production process, technology could be employed combining ordinary multiple planting density adjustment cultivation to achieve the identical performance of energy savings. This took place in the circumstance that the seedlings were transplanted in the density of fully grown plants with no more transplant processing, as long as the two principles during transplanting were followed.

## Author Contributions

KL brought up the idea, designed the system and experiments, modified the manuscript and discussed with reviewers and editor. ZL manufactured the illuminating system, conducted the experiments, analyzed the experiment data and initially formed the manuscript. QY made recommendations, suggestions and modified the manuscript.

## Conflict of Interest Statement

The authors declare that the research was conducted in the absence of any commercial or financial relationships that could be construed as a potential conflict of interest.
